# The Role of Data in Model Building and Prediction: A Survey Through Examples

**DOI:** 10.3390/e20100807

**Published:** 2018-10-22

**Authors:** Marco Baldovin, Fabio Cecconi, Massimo Cencini, Andrea Puglisi, Angelo Vulpiani

**Affiliations:** 1 Dipartimento di Fisica, “Sapienza” Università di Roma, p.le A. Moro 2, 00185 Roma, Italy; 2 Istituto dei Sistemi Complessi, CNR, via dei Taurini 19, 00185 Rome, Italy; 3 CNR-ISC and Dipartimento di Fisica, Sapienza Università di Roma, p.le A. Moro 2, 00185 Roma, Italy; 4 Centro Linceo Interdisciplinare “B. Segre”, Accademia dei Lincei, via della Lungara 10, 00165 Rome, Italy

**Keywords:** models, data, multiscale systems, Langevin equation

## Abstract

The goal of Science is to understand phenomena and systems in order to predict their development and gain control over them. In the scientific process of knowledge elaboration, a crucial role is played by models which, in the language of quantitative sciences, mean abstract mathematical or algorithmical representations. This short review discusses a few key examples from Physics, taken from dynamical systems theory, biophysics, and statistical mechanics, representing three paradigmatic procedures to build models and predictions from available data. In the case of dynamical systems we show how predictions can be obtained in a virtually model-free framework using the methods of analogues, and we briefly discuss other approaches based on machine learning methods. In cases where the complexity of systems is challenging, like in biophysics, we stress the necessity to include part of the empirical knowledge in the models to gain the minimal amount of realism. Finally, we consider many body systems where many (temporal or spatial) scales are at play—and show how to derive from data a dimensional reduction in terms of a Langevin dynamics for their slow components.

## 1. Introduction

It is not an exaggeration to say that models are unavoidable in scientific practice, and that it is impossible to have real science without them. Models are representations and/or abstractions of real phenomena [1,2] that allow for predictions on the phenomena and for understanding the impact of changing some component or parameter of the system. They can be material, such as scaled models of an airplane in a wind tunnel adopted in engineering or simple organisms used in biology (e.g., Drosophila or bacteria in evolutional studies). Other models, the subject of the present review, can be expressed in mathematical terms such as equations or algorithms. Even those very complete and elegant descriptions that we call *theories*, such as classical mechanics or electrodynamics, are nothing but very sophisticated models.

A good starting point for a general discussion about the role of mathematical models for the description of phenomena is *A dialogue on the application of mathematics* written by A. Rényi [3]. The protagonists are King Hieron and Archimedes, whose burning mirrors allowed the Syracusans to sink half of the Roman fleet effortlessly. In the dialogue Archimedes illustrates the applications of mathematics to concrete problems, the central role of models, and the thoughtfulness necessary to master the art of constructing them.

First of all, one can construct many mathematical models for the same practical situation, and one has to choose the most appropriate, that which fits the situation as closely as practical aims require (it can never fit completely). At the same time, it must not be too complicated, but still must be mathematically feasible. These are, of course, conflicting requirements and a delicate balancing of the two is usually necessary. […] You have to approximate closely the real situation in every respect important for your purposes, but lay aside everything which is of no importance for your actual aims. A model needs not to be similar to the modeled reality in every respect, only in those which really count. On the other hand, the same mathematical model can be used to fit quite different practical situations. […] In trying to describe such a complicated situation, even a very rough model may be useful because it gives at least qualitatively correct results, and these may be of even greater practical importance than quantitative results. My experience has taught me that even a crude mathematical model can help us to understand a practical situation better, because in trying to set up a mathematical model we are forced to think over all logical possibilities, to define all notions unambiguously, and to distinguish between important and secondary factors. Even if a mathematical model leads to results which are not in accordance with the facts, it may be useful because the failure of one model can help us find a better one [3].

The point of view of Archimedes was shared by Rosenblueth and Wiener [1] who concluded their paper “on the role of models in science” writing:

The ideal formal model would be one which would cover the entire universe, which would agree with it in complexity, and which would have a one to one correspondence with it. Any one capable of elaborating and comprehending such a model in its entirety, would find the model unnecessary, because he could then grasp the universe directly as a whole. He would possess the third category of knowledge described by Spinoza. This ideal theoretical model cannot probably be achieved. Partial models, imperfect as they may be, are the only means for understanding the universe. This statement does not imply an attitude of defeatism but the recognition that the main tool of science is the human mind and that the human mind is finite.

It is pretty impossible to detail the different procedures which had been followed to build the many models used in science, also because it can be safely claimed that there are not systematic protocols for model building. Possibly a lucky non-trivial exception is given by the framework of classical physics where a fairly clear approach exists. Once the forces are understood, one can write down proper differential equations that may be difficult to solve, but that always allow us to obtain reliable and useful results, for instance by means of qualitative or numerical analysis. Another example is when a reduced model is derived from a more general theory, for instance this the case of the Lorenz’s model [4], which is obtained from a rather crude simplification of fluid dynamics equations. Such a model, in spite of its (apparent) simplicity, played a key role by allowing us to realize that many irregular motions encountered in nature can be due to chaos. This is a clear example of how even a “partial model” can help for understanding natural phenomena.

The above approach is decidedly not applicable to model biological phenomena, medical or economical issues, not to mention social behaviors. In these areas there is nothing like Newton’s or Maxwell’s laws, and therefore a non trivial model can only be achieved either by a pragmatic approach using proper variables that can be guessed only via a good understanding of the subject [5] or from some (often profound) intuition, usually suggested by empirical observations and/or analogies. An instance of the latter is the model of Lotka-Volterra [6,7] which was built in analogy with kinetic theory to describe the evolution of simple ecosystems.

Given the rising pace at which data are produced and accumulated, we think it is timely to briefly review the different approaches to employ available data to build models and predictions of certain phenomena, discussing both classical and modern approaches. To illustrate the possible strategies, we will discuss some specific examples, taken from dynamical systems, the physics of biopolymers and statistical mechanics of many particle systems.

The paper is organized as follows. Section 2 is devoted to a short interlude about the Lorenz and Lotka-Volterra equations, which had an important role in the modeling of natural phenomena and are here presented mainly for historical reasons. Then the discussion will focus on the role of data in the process of building models and formulating predictions. Section 3 is devoted to the use of data and models for the prediction problem. In Section 4 we describe the data driven approach for protein modeling. Section 5 treats the procedures to build Langevin equations, also from data, in the case of systems with fast and slow variables. In Section 6 some general remarks about the relevance of the used variables, the proper coarse graining level, and open problems in the modeling of phenomena using just data.

## 2. An Interlude: The Story of Two Seminal Models

It is instructive to start with a short interlude on the genesis of two models which had a very important role in the history of modeling, and show the role of intuition, as well as mathematics, in the building of a description of natural phenomena. The aim of such models is not a detailed representation of the problem under investigation, but to catch some (qualitative) aspects.

### 2.1. The Lorenz’s Model: How to Obtain Something Interesting with a Crude Assumption

In his famous 1963 paper [4], Lorenz, studying the problem of atmospheric convection, derived a low dimensional model which is one of the first examples of deterministic system with chaotic behavior. Let us briefly recall the used approach. He had to face a rather common problem: Given a nonlinear partial differential equation

(1) $$\partial_{t}\psi \left(x,t\right)=L\left[\psi \left(x,t\right),\nabla \psi \left(x,t\right),\Delta \psi \left(x,t\right)\right]$$

where ψ is a vector field, we want to find a set of differential equations that approximate (1). In the case treated by Lorenz ψ=(u,T), where u and *T* denote the velocity and temperature fields respectively, and the evolution law (1) is the Boussinesq equation which can be considered the “true” description (i.e., the theory) of the system under consideration, i.e., atmospheric convection.

In general, Equation (1) cannot be solved analytically, thus one is forced to resort to a numerical approach. To do this, the first step is to transform Equation (1) into a set of ordinary differential equations. A customary procedure (the so-called Galerkin method) consists in approximating ψ(x,t) in the form

(2) $$\psi \left(x,t\right)=\sum_{n<N}a_{n}\left(t\right)\phi_{n}\left(x\right),$$

where $\left\{\phi_{n}\right\}$ are suitable orthonormal, complete functions. Substituting (2) in (1), one obtains a set of differential equations for the coefficients $\left\{a_{n}\right\}$:

(3) $$\frac{da_{n}}{dt}=F_{n}\left(a_{1},a_{2},..,a_{N}\right)\;\;\;,\;\;n=1,2,\ldots ,N.$$

For the above system to be a good approximation of (1), *N* has to be very large. For instance, in applications in meteorology or engineering, the value of *N* easily reaches $10^{9}$ and even more. It is worth mentioning that in the Galerkin method, from a mathematical point of view, the choice of the functions $\left\{\phi_{n}\right\}$ is largely arbitrary. The unique criterion is that in the limit N≫1 the truncated Equation (3) is a good approximation of the original Equation (1). In order to go beyond simple choices, such as trigonometric functions, if one wants to avoid the use of very large *N* (this in principle is possible if the dimension of the system is not huge) it is necessary to use a “clever” complete, orthonormal set of eigenfunctions, which can be obtained only using some experimental data. An example of such procedure—the proper orthonormal decomposition (POD) [8]—is sketched in the Appendix A.

In his celebrated paper, Lorenz used N=3: In particular, 2 harmonics for the temperature and 1 for the velocity. Its aim was to explore whether such a drastic approximation could reveal some qualitative features of the original problem. His famous, apparently simple, model consists of the following three ordinary differential equations:

(4) $$\frac{dx}{dt}=-\sigma x+\sigma y,\;\;\;\frac{dy}{dt}=-xz+rx-y,\;\;\;\frac{dz}{dt}=xy-bz,$$

where (x,y,z) are proportional to $\left(a_{1},a_{2},a_{3}\right)$, and σ, *b* and *r* are constants related to the properties of the fluid; in particular, *r* is proportional to the Rayleigh number.

It turns out that even the simplified, and physically very crude, Equation (4) cannot be solved analytically. The numerical study performed by Lorenz showed a highly non-trivial behavior, characterized by non-periodic evolutions and, more remarkably, by the property that even very close initial conditions quickly (exponentially fast) evolve in time into very different states. This is the essence of deterministic chaos.

So the importance of Lorenz’s model lies in having shown that it is possible to obtain a chaotic (irregular) behavior even in low-dimension deterministic systems. This result finally led to understand that the complexity of the temporal evolution occurring in turbulent fluids is not a mere superposition of many elementary events (say, many Fourier harmonics), but originates from the nonlinear structure of the equations, which gives rise to chaos [9].

### 2.2. The Lotka-Volterra Model: The Power of the Analogy

Volterra came into contact with data from fishing for the period 1903–1923 gathered by his future son-in-law Umberto D’Ancona. In particular, the data concerned the presence of cartilaginous fish in the catch of three Adriatic ports: Trieste, Venice, and Fiume (now Rijeka).

Volterra simplified in a drastic way the description of marine ecosystem considering only two variables: *x* and *y* representing the number of prey (small fishes) and predators (big fishes), respectively. Then he reasoned by analogy with the kinetic theory: *The big fish “collide” with the small fish and with a certain probability the former eats the latter*, and thus came to identify two differential equations (see below) as a model capable of accounting for D’Ancona’s observations [6].

Some years before, Lotka [7] observed experimentally some patterns in certain chemical reactions, but these patterns turned out to be oscillatory [10]. Volterra, unaware of the Lotka’s work, arrived to the same equations for the dynamics of the two populations:

(5) $$\frac{dx}{dt}=ax-bxy\;\;,\;\;\frac{dy}{dt}=-cy+dxy,\;\;$$

where the constants *a*, *b*, *c*, and *d* are positive. The linear terms are quite transparent: Assuming unlimited food resources, the absence of predators leads to an exponential proliferation of the prey; analogously, in the absence of prey, predators become extinct. In the fish-related case that aroused Volterra’s interest, the non-linear terms have the following interpretation: When both cartilaginous fish and their prey increase, the population transfer from prey to predators also increases.

Despite the simplicity of the model, it is possible to derive a theoretical prediction that is anything but trivial, and in particular the *mathematical explanation* of zoological observations. Suppose the two fish populations are not in equilibrium. Then the abundance of small fish leads to an increase in the population of cartilaginous fish. These, in turn, by hunting will quickly cause a decrease in the population of the other species, until some cartilaginous fish will starve, allowing the repopulation of small fish. And so on, periodically.

Perhaps some readers will have noticed in Equation (5) the (bilinear) structure similar to that of Boltzmann equation for kinetic theory of gases. This is not a mere coincidence: The nonlinear terms were introduced by Volterra noting the analogy between the prey/predator interaction and the impact of two atoms in the kinetic theory while, as we have seen above, Lotka had chemical reactions in mind—in fact, it is the same mechanism.

Once the model based on the Equation (5) has been constructed, we can reason about it in a purely mathematical terms, asking ourselves if it is possible to extend the predictive ability of the model itself. For instance, it is natural to wonder if the model only “works” with algebraic nonlinearities, or if we can consider the case with *N* different species $x_{1}$, …, $x_{N}$. In ecology there is nothing similar to Newton’s mechanics, and therefore such generalizations cannot be sought by relying on first principles. However, the analogy with the Lotka-Volterra model and the consistency with the relevant ecological facts emerge as very natural constraints. Thus, Kolmogorov introduced a generalization of the Lotka-Volterra equations for the N=2, while Smale considered the case with N≥3 for a class of possible equations [11].

Limiting ourselves to quadratic nonlinearities, the obvious generalization of the Lotka-Volterra model is

(6) $$\frac{dx_{n}}{dt}=a_{n}x_{n}\left(1-\sum_{j=1}^{N}b_{n,j}x_{j}\right),$$

where $a_{n}$ is positive for prey (herbivores) and negative for predators; the diagonal terms describe the competition between individuals of the same species, the non-diagonal ones specify the type of interactions between the various species, for instance of parasitic, mutualistic, or prey/predator type.

Summarizing: Data, or rather observations, were fundamental for both Lotka and Volterra, but the brief accounts of their motivations and their arguments for choosing Equation (5) suggest a predominantly heuristic role. Analogy, mathematical intuition and deduction are the main components in the justification of the equations of the model, which both make an effort to show to be *consistent* with fundamental ecological facts. This provides the theoretical basis to derive, this time from the model and therefore mathematically, new conjectures to be submitted to experimental observation. We conclude remarking that generalization of the Lotka-Volterra equations such as (6) are still widely used to study the evolution of ecosystems and in population biology [11].

## 3. The Role of Data and Models for the Prediction

Forecasting the future has always been a desire, and a need, for humans and a natural motivation for science and the development of models. Assuming that the system of interest can be accessed experimentally, having a time record of its evolution, how can we predict its future evolution? The problem so stated is too general and vague. In order to make some precise statement we have to define the classes of system of interest.

Here, we shall restrict our interest to deterministic dynamical systems, whose future is uniquely determined by their present state $x=(x_{1},\ldots ,x_{N})$, where ${\left\{x_{i}\right\}}_{i=1}^{N}$ are all the variables necessary to describe the system state, whose evolution is ruled either by discrete time maps,

(7) x(t+1)=F(x(t)),

or by ordinary differential equations,

(8) $$\frac{dx(t)}{dt}=f\left(x\left(t\right)\right)\;.$$

In this framework, we can distinguish two cases: When we know and can measure the whole state vector x and thus have access to a sequence of its states; when we only have a time record of some scalar quantity related to the system variables.

### 3.1. Prediction When We Know the System State Vector

This case is actually rather infrequent. However, assuming that we are in this fortunate circumstance we have two possibilities. The first corresponds to the ideal scenario in which we know the laws ruling the evolution of the state variables, i.e., we know the right hand side of Equation (7) or (8). The second case is when we do not have such a knowledge.

When the evolution laws are known the main limitation to the possibility of predicting the future is due to the inaccuracy in our knowledge of the present state—*the initial condition*—of the system. Indeed, it is by now well established that the ubiquity of chaos in nonlinear dynamical systems leads to a sensitive dependence on the initial condition. In mathematical terms, this means that even if we precisely know the r.h.s. of Equation (7) or (8), if the equations give rise to a chaotic dynamics, any small uncertainty, ||δx(0)||≪1, on the system state at time t=0 will grow exponentially in time, ||δx(t)||≈||δx(0)||exp(λt), at a rate given by the maximal Lyapunov exponent, λ>0, of the system. In this case, knowing the past evolution of the system does not add much to the prediction, the best we can do is simply to have the most accurate measurement of the state at a given time, i.e., $\left||\delta x\left(0\right)|\right|=\delta_{0}\ll 1$, and then solve the equations to predict the behavior knowing that if we accept an accuracy ||δx(t)||≈Δ on the state, the prediction will be valid up to a time of order [12]

(9) $$T\left(\Delta ,\delta_{0}\right)\approx \frac{1}{\lambda }\ln\left(\frac{\Delta }{\delta_{0}}\right)\;,$$

where the above equation holds under the assumption that not only $\delta_{0}$ but also Δ is small, so that the uncertainty is effectively controlled by the linearized dynamics.

More interesting is the case in which we do not know the equations of motion: In this case we can either hope to build the model, i.e., derive the equations using the time record of the system state and then use it to make predictions (facing the difficulties of the previous case), or attempt a model-free prediction.

Clearly deriving the equations of motion from data records without inputs from theories or intuition, i.e., virtually in an automated way, would be the grand goal of data driven science, i.e., a sort of reverse-engineering from data to infer predictive mathematical models. This would be particularly useful in biophysics and system biology where structured theories from first principles as in physics are basically absent. However, in spite of some interesting attempts in this direction, see e.g., [13], we are still far from this grand goal. Indeed some authors pointed out that the reconstruction of dynamical equations from data is a computationally NP-hard problem [14].

It is however worth mentioning that some inference on the equation of motion can be done when one has some ideas on the possible structure of the equation of motions. For instance, for some specific systems, like networks of nonlinearly coupled oscillators, with a record of the phases evolution it is possible to use optimization methods with some “educated guesses” on the type of interactions (see, e.g., Ref. [15]), or using Bayesian inference [16]. Other interesting approaches, with a wider scope, are based on so called symbolic regression [17], in which the functional form of the terms ruling the interactions among the system variables is not completely preassigned. In a nutshell the idea is to assume a set of building blocks (mathematical structures). As the method requires a search in the huge space of all possible interactions among the variables, it is typically very slow and of limited application. However, we can mention recent improvements based on the idea of sparseness, i.e., on the fact that only a few mathematical structures are represented in certain classes of systems. In this case using appropriate library of mathematical functions (e.g., polynomials of two or three variables, or more elaborated functions) together with powerful machine learning algorithms it has been possible to also reconstruct the dynamics of complicated fluid dynamical systems [18]. We notice that these methods are based on reconstructing the r.h.s of Equations (7) and (8), this means that the time record of the whole system state should be sampled and with time intervals short enough to be able to reconstruct the dynamics, i.e., the time derivative in the case of (8).

A completely different perspective is to attempt predictions by using virtually model-free methods. In this direction, besides a classical approach—based on the so-called method of the analogues, used in many contexts, see e.g., [19,20] for weather forecasting—that will be discussed in the next subsection, which is applicable also when the whole state vector of the system cannot be accessed, we briefly mention a recent promising attempt based on machine learning techniques.

In 2004 Jaeger and Haas [21] proposed a technique known as “reservoir computer”. In a nutshell the idea is to use the time record of the system state, with dimension $D_{in}$, as input of a neural network composed of three elements: An input layer with precisely $D_{in}$ nodes (one for each of the system variables); a nonlinear network with $D\gg D_{in}$ sparsely connected nodes (with appropriate choice of the adjacency matrix of the internal network to ensure some level of activity even in the absence of input, see [21] for details), which are then mapped into an output layer with, again, $D_{in}$ nodes. The network is then trained, using the available time record, by requiring that the output reproduces the input. The latter step is done leaving unchanged the adjacency matrix of the internal network but modifying the weights that map the internal *D* nodes into the output $D_{in}$ nodes, with standard techniques. Once the training is finished, the idea is to use a given state of the system as input—the initial condition—and use the output as new input. This way we have a “new” dynamical system that evolves the state vector of the system of interest. In other words a “model” for the evolution which we do not fully control. However, as shown in Ref. [21], the resulting dynamics predicts very well the system evolution at least for low dimensional systems (e.g., the Lorenz 1969 model). In the last years, the group of Ott has shown that the same idea can be used to predict the evolution of high-dimensional spatio-temporal chaotic systems [22]. Strikingly, in Ref. [22] it has been shown that not only the “new” dynamical system can be used to predict the evolution of the original system but it also provides the correct spectrum of Lyapunov exponents, including the negative ones. Then some extensions of the method have been proposed for very large systems [23], where the idea is to use several internal networks for different portions of the system, or to predict unmeasured variables provided the training step was performed using the whole state vector [24].

### 3.2. Prediction When We Do Not Know the System State Vector

In typical situations we do not know the whole set of variables (not even their number) defining the state of a system. Moreover, even knowing them, in experimental measurements, we usually have access only to a few scalar observables u(t) depending on the state: u(t)=G[x(t)]. In these cases, for not too pathologic function *G*, there exists a powerful technique, based on Takens’ *delay embedding theorem* [25,26], able to reconstruct the phase space. Essentially, these theorems establish that if the system dynamics is effectively *D*-dimensional, where *D* is not necessarily the number of variables of the state space and is not necessarily an integer number (see below), from a long data record of a scalar observable, $u_{k}=u\left(k\Delta t\right)$ with k=1,…,M and Δt the sampling time, we can build a delay coordinate reconstruction, i.e., define a vector in a m−dimensional space

(10) $$U_{k}^{\left(m\right)}=\left(u_{k},u_{k-1},\ldots ,u_{k-(m-1)}\right)\;,$$

which is equivalent, in a sense specified below, to the original one if m≥2[D]+1, where [D] denotes the integer part of *D*. Technically speaking, assuming the original dynamical system to be dissipative, the dynamics will evolve onto a manifold, the attractor, of Hausdorff (fractal) dimension D<N (where *N* is the dimensionality of the state vector), which roughly represents the number of “active” degrees of freedom of the system, below we will give more specifications. Essentially the embedding theorems ensure that the manifold reconstructed by the delay vectors preserves the properties of the dynamical systems that do not change under a smooth change of variables, such as, e.g., the Lyapunov exponents.

The equivalence between the dynamics of the original state vector and the delay coordinate is a very important result. Consider the simple case in which the scalar series ${\left\{u_{k}\right\}}_{k=1}^{M}$ corresponds to the evolution of one of the variables of the system. Then the embedding theorem ensures that it suffices to reconstruct properties of the whole set of variables composing the system. In particular, this means that if we have access, e.g., to two variables, if they belong to the same dynamical system (and thus they are causally dependent) the delay reconstruction made using one of the variable is connected (by a smooth change of coordinate system) to the reconstruction made using the delay vector based on the other variable. For example, using this property in Ref. [27] it was proposed a method to infer causality, in a nutshell one searches for neighbors in one delay reconstruction and study the correlation with neighbors in the reconstruction based on the other variable.

Now let us discuss how one can use a time record to perform predictions (we shall closely follow [28]). In a nutshell the basic idea, in its simplest formulation, can be summarized with the old adage “If a system behaves in a certain way, it will do it again”, which finds its ground in the observation of regularities (periodicity) such as, for instance, the diurnal and seasonal cycles. This idea, together with the belief in determinism (*from the same antecedents follow the same consequents*), it is at the basis of prediction methods. However, as Maxwell argued [29]: *It is a metaphysical doctrine that from the same antecedents follow the same consequents.[…] But it is not of much use in a world like this, in which the same antecedents never again concur, and nothing ever happens twice.[…] The physical axiom which has a somewhat similar aspect is “That from like antecedents follow like consequents.”* These words warn us on the almost exceptional character of periodic behaviors, indeed nowadays we know about the ubiquitous presence of irregular evolutions due to deterministic chaos.

A mathematical formalization of the above idea was due to Lorenz and it is at the basis of the *method of analogues* [19,20], which can be considered as the most straightforward approach to predictability in the absence of a detailed knowledge of the physical laws.

In its simplest implementation, the method works as follows. Assume that the known state x(t) of a process can be sampled at times $t_{k}=k\Delta t$ with arbitrary precision, for considerations on the unavoidable presence of measurement noise we refer to the technical literature on nonlinear time series analysis [30]. In the case in which we only have a partial knowledge of the “true” state, one can use delay vectors (10). In the sequel, for the sake of simplicity of presentation and without losing generality, we can assume that that we have a time series of the system state, sampled at each interval Δt, which is also assumed to be arbitrary but not too short (see, e.g., [30] for a discussion on the proper way to choose the sampling time in practical cases). Given the sequence $x_{k}=x\left(t_{k}\right)$ with k=1,…M, how can we forecast the evolution of $x_{M}$, i.e., predict $x_{M+T}$ at time $t_{M+T}$ (T≥1)? The basic idea is to search in the past $\left(x_{1},x_{2},\ldots ,x_{M-1}\right)$ that state, say $x_{k^{*}}$, most similar to $x_{M}$, and to use its consequents as proxies for the future evolution of $x_{M}$. Mathematically, we require that $|x_{k^{*}}-x_{M}|\leq \epsilon$, and we dub $x_{k^{*}}$ a ϵ-*analogue* to $x_{M}$. We stress that when searching for analogues in the delay reconstructed space one should be aware that if the embedding dimension is not appropriately chosen, a lot of *false* analogues may be present, for a discussion on how to test and avoid this problem see, e.g., [30]. If the analogue were perfect (ϵ=0) the system (being deterministic) would be surely periodic and the prediction trivial: $x_{M+T}\equiv x_{k^{*}+T}$ for any *T*. If it were not perfect (ϵ>0), we could use the forecasting recipe

(11) $${\hat{x}}_{M+T}=x_{k^{*}+T}\;,$$

as *from like antecedents follow like consequents*. For the prediction (11) to be meaningful, the analogue $x_{k^{*}}$ must not be a near-in-time antecedent. A straightforward generalization of (11) is thus to use as a prediction the average over the future evolution of all ϵ-analogues of $x_{M}$ [30].

Once a “good” analogue, i.e., with ϵ reasonably small, has been found, the next step is to determine the accuracy of the prediction. With reference to Equation (11) this means to quantify the evolution of the error $|{\hat{x}}_{M+T}-x_{M+T}|$. In practice, the ϵ-analogue is the present state with an uncertainty, $x_{k}=x_{M}+\delta_{0}$ ($\delta_{0}\leq \epsilon$). Therefore, if the underlying system is chaotic one expects the error to grow exponentially and thus we go back to Equation (9). So that, apparently, the main limit to predictions based on analogues is the sensitive dependence on initial conditions, typical of chaos. But, as realized by Lorenz himself, the main issue is actually to find good (small ϵ) analogues [20]: *In practice this procedure may be expected to fail, because of the high probability that no truly good analogues will be found within the recorded history of the atmosphere.* He also pointed out that, even in the presence of chaos, the main issue with the method relies on the need of a very large data set [19].

In the rest of this section we will illustrate why it is so difficult to find “good” (small ϵ) analogues. We shall begin recalling some basic notions and facts from ergodic theory of dynamical systems that will then be illustrated with a numerical example.

The founding idea of ergodic theory is that the long-time statistical properties of a system can be equivalently described in terms of the invariant (time-independent) probability, μ, such that μ(S) is the probability of finding the system in any specified region *S* of its phase space. If the trajectories of a *N*-dimensional ergodic system evolve in a bounded phase space $\Omega \in R^{N}$, the Poincaré recurrence theorem [31] ensures that analogues exist as it proves that the trajectories exiting from a generic set S∈Ω will return back to such set *S* infinitely many times. The theorem holds for almost all points in *S* except for a possible subset of zero probability. It was originally formulated for Hamiltonian systems, but it can be straightforwardly extended to dissipative ergodic systems provided initial conditions are chosen on the attractor, i.e., on the set $A\in R^{N}$ onto which the system states asymptotically evolve, and “zero probability” is interpreted with respect to the invariant probability, dμ(x), on the attractor. In *N*-dimensional dissipative systems, the attractor *A* has typically a dimension $D_{A}<N$. Slightly more formally, the dimension $D_{A}$ describes the small scale (ϵ≪1) scaling behavior of the probability $\mu \left(B_{y}^{N}\left(\epsilon \right)\right)$ of finding points x∈A which are in the *N*-dimensional sphere, $B_{y}\left(\epsilon \right)$, of radius ϵ around y:

(12) $$\mu \left(B_{y}^{N}\left(\epsilon \right)\right)=\int_{B_{y}^{N}\left(\epsilon \right)}d\mu \left(x\right)∼\epsilon^{D_{A}}\;.$$

when $D_{A}$ is not integer, the attractor and the probability measure on it are dubbed fractal. Typically, $D_{A}$ depends on the point y (i.e., the measure on the attractor is multifractal [12]). For the sake of simplicity, we shall ignore this in the following and assume that there is a single dimension $D_{A}$, i.e., we assume the attractor to be homogeneous.

Poincaré theorem merely proves that a trajectory (almost) surely returns to the neighborhood of its starting point—i.e., the existence of analogues—but does not provide information about the time between two consecutive recurrences—the Poincaré recurrence time. The latter is however crucial to the method of analogues. Indeed, to hope to find an ϵ-analogue of given state one needs the time record of the system state to have a duration of the order of the recurrence time. Another result in ergodic theory, the Kac’s lemma [32], is about the recurrence time. It states that given a set *S* including $x_{0}$ (i.e., the state we are interested in), for an ergodic system, the mean recurrence time of $x_{0}$ relative to *S*, $\langle \tau_{S}\left(x_{0}\right)\rangle$, is inversely proportional to the measure of the set *S* [32], i.e.,

(13) $$\langle \tau_{S}\left(x_{0}\right)\rangle \propto \frac{1}{\mu (S)}\;,$$

where the average is computed over all the states x∈S according to the invariant measure. Now taking $S=B_{x_{0}}^{N}\left(\epsilon \right)$ and using (12) yields

(14) $$\langle \tau_{\epsilon }\left(x_{0}\right)\rangle ∼\epsilon^{-D_{A}}\;.$$

Therefore, if we require ϵ to be very small and it happens that $D_{A}$ is large the average recurrence time becomes indeed huge.

As the above description is somehow formal, it is useful to illustrate it with a specific example. We consider the following nonlinearly coupled ordinary differential equations, first proposed by Lorenz in 1996 [33]:

(15) $$\frac{dX_{n}}{dt}\;=\;X_{n-1}\left(X_{n+1}\;-\;X_{n-2}\right)-X_{n}+F\;,\;n\;=\;1,\ldots ,N\;,$$

with periodic boundary conditions ($X_{N\pm n}=X_{\pm n}$). The variables $X_{n}$ may be thought of as the values of some atmospheric representative observable along the latitude circle, so that Equation (15) can be regarded as a one-dimensional caricature of atmospheric motion [33]. The quadratic coupling conserves energy, $\sum_{n}X_{n}^{2}$. In the presence of forcing *F* and damping $-X_{n}$, the energy is only statistically conserved. The motion is thus confined to a bounded region of $R^{N}$. Moreover, dissipation constrains the trajectories to evolve onto a subset—the attractor—of this region possibly with dimension $D_{A}<N$. The dynamical features are completely determined by the forcing strength *F* and the system dimensionality *N*. For F>8/9 and N≥4 the system is chaotic [34].

Assuming ergodicity, Poincaré theorem holds with respect to the invariant measure and for any generic state we should expect analogues to exist for any ϵ. Let us now count them. Being interested in typical behaviors and not just in the properties around a specific state we select *r* states ${\left\{X_{⋆}^{k}\right\}}_{k=1}^{r}$ along a given trajectory of (15), well spaced in time to sample the attractor, and we compute the average fraction of their ϵ-analogues as

(16) $$C_{r,M}\left(\epsilon \right)=\frac{1}{Mr}\sum_{k=1}^{r}\sum_{j=1}^{M}\Theta (\epsilon -|X_{j}-X_{⋆}^{\left(k\right)}\left|\right)\;.$$

Practically, as we know the evolution laws (15), instead of looking at the backward time series of the reference states, we can select the ${\left\{X_{⋆}^{k}\right\}}_{k=1}^{r}$ and look at their forward ϵ-analogues.

Now we notice that the average fraction of ϵ-analogues Equation (16) is nothing but the fraction of time the trajectory spends in a sphere of radius ϵ centered around the reference states. Thus, for large *M*, as a consequence of ergodicity, $C_{r,M}\left(\epsilon \right)$ is simply the average probability to visit these ϵ balls around the reference points. Therefore, with the assumption that only the dimension $D_{A}$ characterizes the system, for sufficiently large *M* and small ϵ, Equation (12) implies $C_{r,M}\left(\epsilon \right)∼\epsilon^{D_{A}}$. Actually, the quantity $C_{r,M}\left(\epsilon \right)$ is a numerical approximation of the so called correlation integral introduced by Grassberger and Procaccia [35] to compute the correlation dimension, $D_{2}$, of strange attractors emerging from dissipative chaotic systems. Here the assumption of homogeneity implies $D_{2}\approx D_{A}$. We notice that accounting for the heterogeneity is a mere technical complication which does not change the main points of this section.

**Figure 1:**
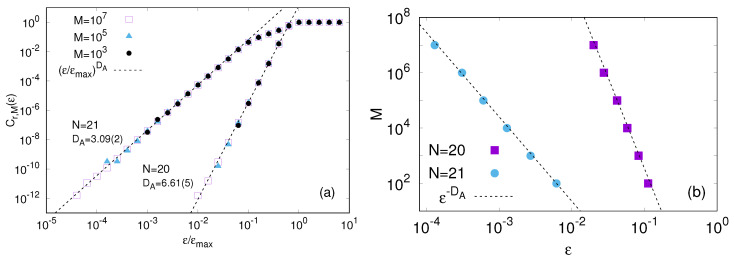
(**a**) $C_{r,M}\left(\epsilon \right)$ vs. $\epsilon /\epsilon_{\max}$ for F=5, N=20 and N=21; the reference states are r=1000 and different values of *M* ranging from $10^{3}$ to $10^{7}$ are considered. The solid lines are the fits of the data assuming $C_{r,M}\left(\epsilon \right)∼\epsilon^{D_{A}}$; (**b**) Number of data points *M* vs $\epsilon_{min}/\epsilon_{max}$ vs. *M*. The dashed lines are the fits of the data by means of relation (18).

In Figure 1 we show $C_{r,M}\left(\epsilon \right)$ obtained with $r=10^{3}$ reference states and different lengths *M* of the time series, from $10^{3}$ to $10^{7}$. When the degree of similarity ϵ becomes larger than the attractor size, say $\epsilon_{\max}$, the fraction $C_{r,M}\left(\epsilon \right)$ saturates to 1. Therefore, we normalize the degree of similarity by $\epsilon_{\max}$. As for the dynamics (15), the forcing is fixed to F=5 and we consider two system sizes N=20 and N=21. In both cases the system is chaotic. The solid lines in Figure 1 indicate that for $\epsilon \ll \epsilon_{\max}$ the probability to find an analogue is fairly well approximated by the expected power law $C_{r,M}\left(\epsilon \right)∼\epsilon^{D_{A}}$. Fitting the data with this expression, we find $D_{A}≃3.1$ and $D_{A}≃6.6$ for N=21 and N=20, respectively. Practically, this difference in $D_{A}$ means that the probability to find ϵ-analogues with N=20 becomes about $\epsilon^{3.5}$ times smaller than with N=21.

Slightly changing the viewpoint, we observe that $C_{r,M}\left(\epsilon \right)$ relates to the Poincaré recurrence times we mentioned above. To understand the latter point, fix r=1, i.e., consider the analogues to the state $x_{M}$ in a sequence $x_{1},\ldots ,x_{M}$ sampled at each Δt and denote their number with M(ϵ). Clearly, the average time interval, $\bar{\tau_{\epsilon }}\left(x_{M}\right)$, between two consecutive ϵ-analogues of $x_{M}$ is

(17) $$\bar{\tau_{\epsilon }}\left(x_{M}\right)=\frac{(M-1)\Delta t}{M(\epsilon )}=\frac{\Delta t}{C_{1,M}\left(\epsilon \right)}\propto \epsilon^{-D_{A}}\;,$$

where we used that $C_{1,M}\left(\epsilon \right)=M\left(\epsilon \right)/\left(M-1\right)$, being it the fraction of ϵ-analogues. Thus $C_{r,M}\left(\epsilon \right)$ is simply a further average of such times over different balls of radius ϵ centered on different reference states. Clearly, in order to find an ϵ-analogue we must require the number of points *M* to satisfy $M\Delta t\geq \bar{\tau_{\epsilon }}\left(x_{M}\right)$, that from (17) implies $M\geq 1/C_{1,M}\left(\epsilon \right)$ and, given the hypothesis of homogeneity of the attractor we have $C_{1,M}\left(\epsilon \right)∼C_{r,M}\left(\epsilon \right)∼\epsilon^{-D_{A}}$, we can realize that the minimum length of the time series is

(18) $$M∼{\left(\frac{L}{\epsilon }\right)}^{D_{A}}\;\;,$$

*L* being the typical excursion of each component of x, used to nondimensionalize the expression.

Equation (18) implies that, at least in principle, the method can work for deterministic systems having an attractor of finite dimension provided the time series is suitably long. However, the exponential dependence on $D_{A}$ in Equation (18) imposes, upon putting the numbers, too severe constraints. For instance in Figure 1b, we show how the number of data-points *M* scales with distance between a reference point and its best analogue ($\epsilon_{\min}$). We see that for $\epsilon_{\min}/\epsilon_{\max}=10^{-2}$ a sequence of $10^{2}$ points is sufficiently long in the case N=21 ($D_{A}\approx 3.1$) while, on the contrary, even $10^{7}$ points are not yet enough in the case N=20 ($D_{A}\approx 6.6$), the rationale for this difference is Equation (18). We notice that the counter-intuitive inequality $D_{A}\left(N=21\right)<D_{A}\left(N=20\right)$ is a peculiar detail of the considered model [34]. Generally, $D_{A}$ is expected to increase with *N* [12]. The values of *N* and *F* here used are motivated to emphasize the importance of the effective number of degrees of freedom that, in general, is not trivially related to (and can be much smaller than) the number of variables *N*.

We stress that the necessity of an exponentially large (with $D_{A}$) amount of data constitutes a genuine intrinsic difficulty of every analysis based on time series without any guess on the underlying dynamics. Such a difficulty is not a peculiarity of the method of analogues, but is inherent to all methods based on the occurrence frequency of sequences of states to estimate the average of observables. The problem arises whenever one needs to collect enough recurrences, including the Grassberger and Procaccia [35] technique or the method to infer causality [27]. Though these considerations may sound trivial, it is worth recalling that, in the ’80s, when nonlinear time series analysis started to be massively employed in experimental data analysis, the limitations due the length of the time series were overlooked and a number of misleading papers appeared even in important journals [36].

Summarizing, in virtue of (18), when $D_{A}≳5-7$ the number of data-points required for finding good analogues is prohibitive. This sounds very pessimistic for predictions from data, it is worth concluding with some words of optimism. Indeed, the problem of finding the analogues may be mitigated in the presence of a multiscale structure, where the vector state x can be decomposed into a slow component X which is also the “largest” one, and a fast component y “small” with respect to X (i.e., $y_{\mathrm{rms}}\ll X_{\mathrm{rms}}$). In such cases, provided that the slow components can be described in terms of an “effective number” of degrees of freedom much smaller than those necessary to characterize the whole dynamics, mediocre (referred to the whole system) analogues can be used to forecast at least the slower evolving component.

We illustrate this point by considering a variant of (15) introduced by Lorenz himself [33] to discuss the predictability problem in the atmosphere, where indeed a multiscale structure is present. The model reads

(19) (dXndt=Xn−1(Xn+1−Xn−2)−Xn+F−hcb∑k=1Kyk,n

(20) (dyk,ndt=cbyk+1,n(yk−1,n−yk+2,n)−cyk,n+hcbXn,

with n=1,…,N, k=1,…,K and boundary conditions $X_{N\pm n}=X_{\pm n}$, $y_{K+1,n}=y_{1,n+1}$ and $y_{0,n}=y_{K,n-1}$. Equation (19) is the same as (15) but for the last term which couples X to y. The variables y evolve with a similar dynamics but are *c* times faster and *b* times smaller in amplitude. The parameter *h*, set to 1, controls the coupling strength. In geophysical fluid dynamics the variable X and y represent the synoptic and convective scales, respectively [33].

**Figure 2:**
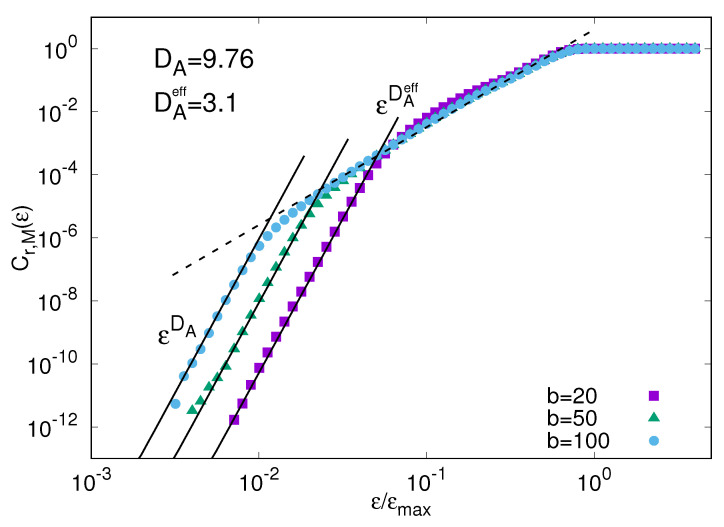
$C_{r,M}\left(\epsilon \right)$ vs. $\epsilon /\epsilon_{max}$ for model (19) and (20) computed for three scale separations *b* (as labeled) holding the other parameters fixed at h=1, c=10, F=10, N=5 and K=10. The dashed line has slope ≈3.1 while the solid lines have all the same slope ≈9.76. The quantity (16) has been computed with $r=10^{3}$ and $M=10^{7}$.

Figure 2 shows the fraction of ϵ-analogues $C_{r,M}\left(\epsilon \right)$ for the system (19) and (20) obtained by assuming that a long record, $M=10^{7}$, of the whole state of the system x(t)=(X(t),y(t)) can be measured. In this example the time scale separation is fixed to c=10 and the fast component y is b=20,50, and 100 times smaller than the slow one X. The phase-space dimensionality is 55, with N=5 slow and K=10 fast degrees of freedom for each slow one. The attractor dimension of the whole system $D_{A}$ is rather large ($D_{A}\approx 10$). However, for $\epsilon /\epsilon_{\max}>O\left(1/b\right)$ a second power law, $C\left(\epsilon \right)∼\epsilon^{D_{A}^{\mathrm{eff}}}$ with $D_{A}^{\mathrm{eff}}\approx 3<D_{A}$, appears defining a sort of “effective dimension at large scale”.

Therefore, if we are interested in predicting the slow evolving component of the system, provided it is described by a relatively low number of effective degrees of freedom, as here, we can exploit the mediocre analogues (i.e., the ϵ-analogues with $\epsilon /\epsilon_{\max}>O\left(1/b\right)$). Moreover, it is reasonable to expect that the prediction error related to mediocre analogues grows in time as $∼\epsilon e^{\lambda (\epsilon )t}$ where λ(ϵ) can be much smaller than the Lyapunov exponent $\lambda_{1}$ (indeed as shown in Ref. [37] $\lambda \left(\epsilon \right)\approx \lambda_{1}/c$). This implies that slow variables can be predicted over longer term than the whole state of the system, as already realized by Lorenz [33]. We remark that the above example is a very simplified idealization of a multiscale system and something much more complicated may happen, see e.g., [38].

We conclude this Section by noticing that unlike the Lorenz 1963 (4), the 1996 models (15), (19) and (20) were not obtained by means of an approximation of hydrodynamic equations, but have been built just using the type (quadratic) of nonlinearities and the conservation laws (i.e., of energy in the inviscid limit) of the original system. They represent toy models which preserve crucial aspects of the original problem with the advantage of simplifying the numerical approach. For instance, Equations (19) and (20) is a playground to understand the dynamics of systems at very different time and spatial scales. In this class of models we mention the shell models for turbulence which have been built to “mimic” the Navier-Stokes equations and allowed for understanding (even quantitatively) many aspects of the energy cascade in turbulence [12,39].

## 4. Protein Modeling: An Example of Data-Driven Approach

The impressive progress undergone by Molecular Biology in the last decades produced a wealth of knowledge and accurate data around protein molecules. All this body of information needs to be rationalized into theoretical frameworks enabling interpretation and prediction of elementary biological processes. In this context, the study of proteins well illustrates how models can be built upon and improved by using experimental data to address more challenging tasks.

Proteins are biopolymers performing or assisting a myriad of biological processes; to become active they need to assume a specific three-dimensional structure: The native or active state (NS). Under physiological conditions proteins fold spontaneously into the NS in a way that is mainly encoded by the sequence of aminoacids [40], and the accurate prediction of the structure from the sequence is a longstanding issue of Molecular Biology known as *protein folding problem* [41]. Computational methods based on clever modeling are the primary tools to assist and complement experimental research in solving the problem.

Before illustrating the key elements of protein models, it is necessary to understand which is the basic feature of a good model. This arises from the comparison between natural proteins and random heteropolymers [41], in fact proteins are known to be anything but random objects.

**Figure 3:**
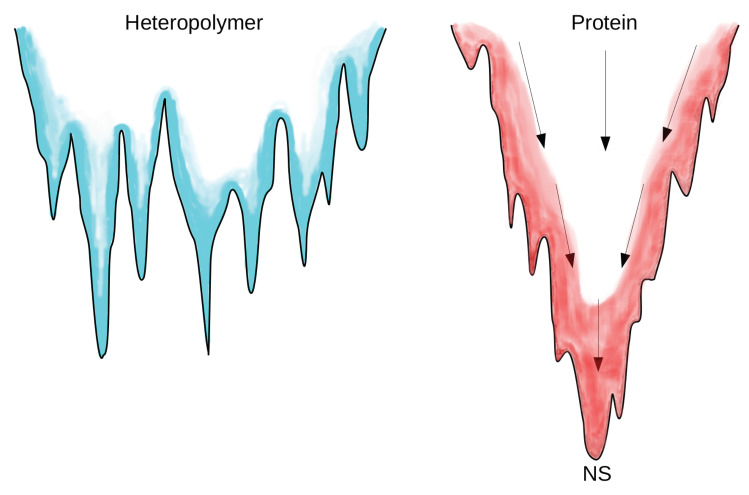
Sketch of the energy landscapes: Glassy-like for random heteropolymers (**left**) with several deep minima and high barriers and funnel-like for proteins (**right**) with the native state (NS) occupying the “unique” minimum.

The difference between proteins and heteropolymers clearly emerges from the structure of their respective energy landscape [42,43], see Figure 3. The energy landscape of random heteropolymers is studded with many degenerate minima, each one representing the end-point of the folding process. The trapping action of such deep metastable minima slows down the folding process. In contrast, protein-like landscapes have been “sculpted” by evolution in a funnel-like shape, where NS is placed at the bottom, Figure 3. The protein, following the funnel, experiences a simultaneous decrease of entropy and energy so that no high barriers emerge along the pathway to the NS. Therefore, the funnel confers thermodynamic stability and fast accessibility to the NS.

The landscape picture strongly suggests that protein models should reproduce a funnel-shaped energy landscape with a moderate amount of frustration.

A general expression of the potential energy of classical physics-based all-atom model consists of various interaction terms [44]

(21) $$U=U_{bond}+U_{angle}+U_{dihe}+U_{vdw}+U_{elec}$$

**Figure 4:**
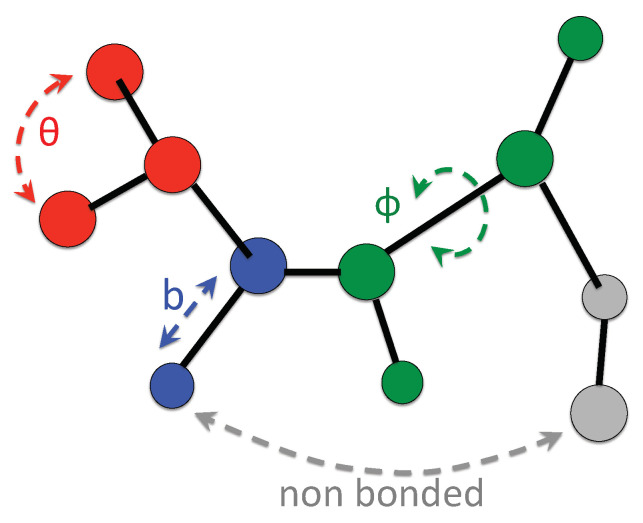
Typical interactions considered in a all-atomistic force field. Atoms of a molecules interact by simple springs, in lengths and angles (bonded contributions), and via long range forces (non bonded contributions). In a mechanical view the forces determine the spatial arrangements of atoms an their dynamics.

The first three of them, so-called “bonded” terms, describe the principal structural deformations (sketched in Figure 4) due to bond stretching (blue), bending (red), and torsion for a rotation about certain dihedral angles (green). The last two terms, referred to as “non-bonded” interactions, describe dispersion and repulsion effects (Lennard-Jones term) and electrostatic interactions.

Despite its conceptual simplicity, the energy function (21) presents two big practical problems when applied to the simulation of real systems. First, for proteins of medium and large size, it involves many atoms of different species with a undesired proliferation of terms and parameters. Second, both realistic parameters and units need to be assigned. This step, generally referred to as the *parameterization of the force-field*, is rather delicate if one requires maximal applicability of the model (transferability). A discussion of these issues is beyond our purpose, here we limit ourself to mention the general strategy. Parameters are optimized to match the increasing body of experimental information: molecular geometries, thermodynamics data and other spectroscopic data. In particular, vibrational spectra of small molecules, detected by infrared radiation and Raman scattering [45], allow for obtaining the force constants and inter-atomic distances of bonded interactions (terms 1,2,3 in Equation (21)). If the measured vibrational frequency of a specific bond is ω, in the model, the strength of that interaction will be set to $k≃\omega^{2}M$, with *M* being the reduced mass of the atoms involved.

Non-bonded interactions can be obtained by the Boltzmann-Inversion method [46]. Suppose, for instance that a scattering experiment sampled the pair-correlation function $g_{ij}\left(r\right)$ between atoms *i* and *j*, then it is natural to assign them the interaction potential,

$$u_{ij}\left(r\right)=C-k_{B}T\ln\left[g_{ij}\left(r\right)\right]\;,$$

where the potential is known up to an additive constant *C*. The above formula stems from the *reversible work theorem* [46], a general result of Statistical Mechanics proving that the pair correlation function of an equilibrium system satisfies the relation

$$g_{ij}\left(r\right)=\exp\left\{-\beta w_{ij}\left(r\right)\right\}$$

where $w_{ij}\left(r\right)$ is the reversible work (free energy) spent to bring the tagged particles i,j from an infinite separation to a distance *r*. This inversion formula can be also applied recursively from an initial guess of potential to improve the potential estimate [47].

In practice, we are in the position to know many elementary rules driving a chain of aminoacids into the native structure, but our algorithmic implementation of the model (21) is currently so inefficient that only reproduces folding events of small protein molecules. Real folding processes of larger structures are still prohibitive to all-atom simulations.

Therefore, a clever strategy of simplifications is needed which brings to the building of Coarse grained (CG) protein models [48,49,50,51]. A CG procedure basically tries to keep a similar form of the all-atom force field (21) while operating a drastic decimation of degrees of freedom to reduce the computational load at the price of losing resolution. In this respect, coarse graining means that groups of atoms are joined together to form “pseudo-atoms” (also named “united-atoms”) which work as centers of interaction in the new simplified model. This procedure, which to preserve the phenomenology must be not too invasive, requires re-definition and re-parameterization of ranges and intensities of the interactions among pseudo-atoms.

The definition of the CG-model interactions follows the same strategy discussed for the all-atomistic force fields, the simplification arises from the reduced number of parameters, however the conceptual difficulty remains. For CG-models there is the additional advantage that the parameterization can also be performed by using the results of the full-atom simulations. This can be addressed by the powerful techniques of inverse-problems and statistical inference, such as max-entropy and maximum likelihood principles [52].

Below we illustrate two CG-approaches that use only protein structural information to derive reasonable interactions among aminoacids. In this respect they constitute examples of models uniquely driven by the empirical knowledge.

### 4.1. Knowledge Based Approach

The accuracy of experimental techniques allows protein databases to be updated at an impressive rate with new experimentally determined structures. This rich information can be exploited to “learn” empirical interactions among aminoacids, via a statistical analysis that looks for recurrent folding rules over a large number of known protein structures.

Tanaka and Scheraga [53] suggested to use a coarse-grained potential obtained from the frequency of aminoacid contacts observed in a set of crystal structures of proteins. A contact means that two aminoacids are neighbors in a given structure, and this proximity is interpreted as the presence of an interaction. Accordingly, the interaction energy between two types of amino acids, a,b, can be assigned as

$$\epsilon_{ab}=-k_{B}T\ln\left[\frac{N_{ab}}{N_{ab}\left(0\right)}\right]$$

where $N_{ab}$ is the observed frequency of the contact of the couple a−b in the representative database. While $N_{ab}\left(0\right)$ denotes the expected frequency of that contact in a chosen reference state, that is a set of disordered (i.e., non-native) conformations where that contact is only “randomly” established. Therefore meaningful interactions emerge from the comparison between the probability of finding them in native-like structures and the probability of occurrence in the reference state of non-native conformations. Of course, the quality of the results depends on the “good choice” of both database and reference state, for this reason, their optimal selection is the subject of debate and much investigation.

The knowledge-based interaction parameters are rather general as they not only incorporate many effects acting between atoms, such as electrostatic, van der Waals forces, etc., but also the influence of the surrounding solvent.

It is interesting to remark that the principle for generating empirical potentials is equivalent to the methods of analogues discussed in Section 3.2. In the former, recurrent regularities are searched in the database of structures, while the latter searches for recurrences in the time series of events.

### 4.2. Structure Based Models

The concept of funnel-like landscape (Figure 3) is carried to extremes in the structure based modeling, originally introduced by Gō and coworkers [54]. These models are built up in such a way that energy function attains its minimum on the coordinates of the crystallographic structure of the NS. Therefore the experimental data enter crucially in the model definition. A simple way to achieve that NS is a minimum is by introducing the notion of native interactions, or *contacts*. Two atoms are said to form a native contact if in the NS they are neighbors, i.e., if their native distance $R_{ij}<R_{c}$ for a chosen $R_{c}$. We assume that only atoms forming native contacts interact attractively, whereas the others repel each other via an excluded-volume potential. As in this framework a decrease in energy can only be achieved through an increase in the fraction of native contacts, the Gō-model minimizes frustration, assuring a fast and correct arrival to the NS.

A popular Gō-like force field was introduced by Clementi et al. [55] who proposed for a *N*-beads system the potential-energy:

$$\begin{array}{ccc} \Phi_{G\bar{o}} & = & \sum_{i=1}^{N-1}\frac{k_{p}}{2}{\left(r_{i,i+1}-R_{i,i+1}\right)}^{2}+\sum_{i=1}^{N-2}\frac{k_{\theta }}{2}{\left(\theta_{i}-\Theta_{i}\right)}^{2}+\\ & & \sum_{i=1}^{N-3}\left\{k_{\varphi }^{\left(1\right)}\left[1-\cos\left(\varphi_{i}-\Phi_{i}\right)\right]+k_{\varphi }^{\left(3\right)}\left[1-\cos\left(3\varphi_{i}-3\Phi_{i}\right)\right]\right\}+V_{nb}\left(r_{ij}\right)\;. \end{array}$$

The first term, that enforces chain connectivity, is a stiff harmonic potential between consecutive residues allowing only small fluctuations of the bond lengths $r_{i,i+1}$ around their native values $R_{i,i+1}$. Likewise, the elastic potential in θ allows only small fluctuations of the bending angles $\theta_{i}$ around their native values, $\Theta_{i}$. The native-like secondary structure (helices, beta-sheets) is enforced by a potential in the dihedral angles. Each $\varphi_{i}$ angle, identified by four consecutive beads, characterizes the local torsion of the chain. Again, $\Phi_{i}$ denotes the value of the *i*-th angle in the native structure. Finally, the long-range potential $V_{nb}$, which favors the formation of the correct native tertiary structure by promoting attractive interactions, is the two-body function

$$\begin{array}{c} V_{nb}\left(r_{ij}\right)=\epsilon \left\{\begin{array}{cc} 5{\left(\frac{R_{ij}}{r_{ij}}\right)}^{12}-6{\left(\frac{R_{ij}}{r_{ij}}\right)}^{10} & R_{ij}\leq R_{c}\\ \frac{10}{3}\;{\left(\frac{\sigma }{r_{ij}}\right)}^{12} & R_{ij}>R_{c}\;. \end{array}\right. \end{array}$$

Therefore, the interaction between aminoacids i−j is attractive when their distance in the native structure, $R_{ij}$, is below a certain cutoff, $R_{c}$, otherwise the aminoacids repel each other via a soft-core σ. It means that the system gains energy as much as its conformation is close to the NS. A unique parameter ϵ sets the energy scale of the force field and the other parameters introduced above are the typical ones used in similar Gō-type approaches, see e.g., [55,56].

This philosophy may seem artificial, as the folding problem is somehow reversed, because the sequence is neglected, and the NS becomes not the final goal but the root of the model. However, a number of empirical evidences supports the use of Gō-models, by underscoring the importance of the topology of the NS, which is encoded by the adjacency matrix representing the network of native contacts. In particular, proteins with different sequences exhibit similar folding mechanisms if they have similar native and transition states. Moreover, simple topological parameters characterizing the complexity of the NS were found to correlate well with the folding rates of small globular proteins [57,58], again showing the relevance of the native structure.

Gō models can be successfully used to sample the Transition State Ensemble, which is the collection of protein conformations that have the same probability (=1/2) either to refold in the NS or to unfold. This knowledge allows the computation of folding rates and the understanding of folding mechanisms for globular proteins [55,56,59,60]. Moreover, since Gō-like models by construction well describe the NS geometry, they offer the opportunity to study the stability of structures during the unfolding [61,62].

The main criticism raised to the Gō-like philosophy is the principle of minimal frustration, which leads to the absence of non-native interactions. Indeed, studies pointed out the relevance of energetic frustration in real proteins because non-native contacts may form in the transition state without being present in the NS [63]. However, the frustration due to non-native contacts is not always deleterious, but seems to have certain relevance in favoring the folding process. At first glance, the introduction of increasingly larger non-native energies is expected to slow down the folding process, however Plotkin and Clementi [64] reached the counter intuitive conclusion that a moderate amount of frustration reduces the free energy barriers and increases folding rates.

Another criticism of Gō models concerns the exclusion of sequence effects. In Gō models the folding is driven only by the topology of the NS, but it is the sequence that determines the topology. The precise role of the sequence in folding remains to be understood. A common strategy to account for sequence effects is by using heterogeneous contact energies that may be chosen using different criteria again based on structural information [65,66].

We conclude this brief survey on CG protein modeling with the final consideration “whether CG models will stand the test of time” as they may appear just as computational tricks to by-pass the limitations of current computational resources. This idea is misleading, as the ability of these models to correctly reproduce some experimental patterns shows that not all the molecular degrees of freedom are equally important. In other words, the CG approaches are able to single out the relevant driving forces among the multitude of chemical details of macromolecules. This is why CG models will remain precious conceptual tools for the study of macromolecular systems even when the advances in computer science will make atomistic simulations feasible on biologically relevant timescales.

## 5. Langevin Equation: When a Multiscale Structure Helps

Several problems in science are characterized by the presence of very different time scales. Among the most important examples, we mention protein folding and climate: for proteins, the time scale of the vibration of covalent bonds is $O(10^{-15})$ s, while the folding time can be of the order of seconds; in the case of climate, the characteristic times of the involved processes vary from days (for the atmospheric phenomena) to $O(10^{3})$ yr for the deep ocean flows and ice shields. The necessity of treating the “slow dynamics” in terms of effective equations is both practical (even modern supercomputers are not able to simulate all the relevant scales involved in certain difficult problems) and conceptual: Effective equations are able to catch some general features and to reveal dominant ingredients which can remain hidden in the detailed description. The study of such multiscale problems has a long history in science, and some very general mathematical methods have been developed [67,68,69], whose usage, however, is often not easy in non-idealized situations.

Historically, the theory of Brownian motion represents perhaps the first example of multiscale physical modeling. In their studies Einstein, Smoluchowski, and Langevin recognized the multiscale structure intrinsic in the movement of a particle of ∼1 μm size suspended in a fluid made of molecules of ∼1 Å size, and exploited such a structure to achieve an effective theoretical model. The Brownian motion model (simple diffusion) deduced by Einstein and Smoluchowski has become the first brick for the future development of the theory of stochastic processes [70,71]. With a different spirit but similar phenomenological arguments (the Stokes law), and statistical assumptions (thermal equilibrium of the colloidal particle with the liquid), Langevin introduced his celebrated stochastic differential equation [72,73] which—after more than a century—stands as an unrivaled paradigm for small systems displaying smooth slow dynamics superimposed with observable fast fluctuations. Most importantly, a fundamental modern wave of non equilibrium statistical mechanics, that is stochastic thermodynamics, is founded upon the Langevin model [70,74,75].

A natural question is the possibility to derive the Langevin Equation (LE) in a non phenomenological way, i.e., starting from the dynamics of large systems [76]. Unfortunately, there are just few cases where it is possible to use such a desirable approach [67]. One situation is the motion of a heavy particle in a diluted gas: In such a case, using an approach going back to Smoluchowski, with a statistical analysis of the collisions among the heavy particle and the light gas particles, it is possible to determine the viscous friction [77,78]. A complete derivation, including the shape of the noise term, has been also obtained as a perturbative expansion of the Lorentz-Boltzmann equation by van Kampen [79], repeated in a similar form for granular gases [80]. A mathematical account of this procedure can be found in [81]. There is also another large class of systems where it is possible to obtain a LE in an analytical way: Harmonic chains with a heavy particle of mass *M* and *N* light particles of mass m≪M [82,83,84]. In such a case, the linearity of the dynamics allows for an explicit solution and then the possibility to find the LE for the heavy particles in the limit m/M≪1 and N≫1.

As far as we know there are no other clearly distinct cases where it is possible to derive a LE starting from a deterministic mechanical model of the whole system. It is certainly desirable to have the possibility to write down a LE for a heavy particle also in systems different from the well known cases above discussed. Approximate derivations for the case of non-linear forces [85] and for cases near non-equilibrium stationary processes [86] have also been recently considered. A particularly interesting test-case is that of systems where the Hamiltonian has a non-standard kinetic term, i.e., non-quadratic in the momentum, leading to non-trivial properties such as negative absolute temperature [87,88,89,90], a possibility recently verified also in experiments [91].

In the following we discuss a very different approach, that is a practical procedure to build a Langevin equation from a long time series of data from experimental or numerical results. Up to our knowledge a similar procedure has been applied, previously, to many model systems [92] but only recently some of us have applied it to Hamiltonian systems, including systems displaying negative temperatures [90]. The procedure discussed here does not include a preliminary check for the validity of a LE description, it blindly assumes such a validity: Of course one then has to wonder in a critical way if such an assumption is correct, or at least, consistent. In the large category of multiscale phenomena, there are different physical ingredients which can provide a justification for a LE description. In many cases—in analogy with what discussed in the above examples—the necessary separation of time scales is guaranteed by the existence of a massive (slow) degree of freedom with respect to the “background”, i.e., the aforementioned condition M≫m. Possible exceptions where such a separation of masses can be insufficient for a LE is discussed in [90]. In real situations one simply assumes the existence of two classes of variables (slow and fast) with well separated time scales and tries to reconstruct a LE for the slow variables. A posteriori, the quality of such an initial assumption can be verified by comparing the original data with the results of the model [68,69,92,93,94]. Some authors have discussed a general mechanism justifying the scale-separation assumption, within a dynamical systems approach [95,96].

We briefly illustrate the LE reconstruction protocol. The basic assumption for this procedure is that the slow variable, say “*v*”, can be described by a Ito-Langevin equation with the shape

(22) $$\frac{dv}{dt}=F\left(v\right)+\sqrt{2D(v)}\eta \left(t\right)$$

where η is a white noise with 〈η(t)〉=0 and $\langle \eta \left(t\right)\eta \left(t^{\prime }\right)\rangle =\delta \left(t-t^{\prime }\right)$. The function F(v), as well as D(v), can be obtained from a long time series of the values of v(t) at time intervals Δt. Being

Δv(t)≡v(t+Δt)−v(t)

we have for consistency with (22) that

(23a)F(v)=limΔt→01Δt〈Δv(t)|v(t)=v〉(23b)D(v)=limΔt→012Δt〈Δv(t)−ΔtF(v)2|v(t)=v〉.

Assuming stationarity, the quantities on the r.h.s. do not depend on time and they can be estimated as temporal averages, for several choices of Δt. By extrapolating their dependence on the time interval in the limit Δt→0, one can infer a good approximation for the drift and the diffusivity.

Of course the practical evaluation of such limits is not completely straightforward, since it involves a careful analysis of the relevant time-scales of the process. In particular, the approximation of the dynamics of a deterministic process as a LE cannot be valid at any arbitrarily small time-scale, but only for Δt larger than a certain threshold (sometimes called the “Markov-Einstein time” [92]): the limit Δt→0, therefore, must be interpreted in a proper physical way.

In several contexts the method has been applied also to Markovian processes in which the independent variable is a spatial coordinate, instead of time *t*: This is the case, for instance, of the reconstruction of rough surfaces performed in Ref. [97]. In that work the height h(x) of a copper film deposited on a polished Si(100) substrate at a certain time has been studied as a function of the spatial coordinate *x*. As a first step, the markovianity of the stochastic process h(x) has been carefully checked; then, the described method has been performed considering a long data series and the reconstructed surface has been compared to the original one, finding a good agreement. Another class of processes which can be treated with this method is that of quantities that depend on the observed temporal (or spatial) scale, e.g., in turbulence analysis [98]. Once the Markov property of the process has been verified, the procedure can be exploited in a completely analogous way.

In principle a slight modification of the method could also be applied to discontinuous stochastic processes, and a generalized LE with Lévy noise could be inferred [99]. In these cases the first step is the determination of the Lévy stability parameter α∈(0,2], which can be measured from long time-series by exploiting the relation:

(24) $$\ln\langle \left(|\Delta v(t)-\Delta tF(v)|\right)|v\left(t\right)=v\rangle =\frac{1}{\alpha }\ln\Delta t+C\left(\alpha \right)\;.$$

One can evaluate the l.h.s. as a temporal average for several values of Δt and then infer α as the inverse of the slope.

Further examples are discussed in some detail in Ref. [92], where a complete illustration of the method is given and its widespread application fields are overviewed.

To conclude we give a brief demonstration of the power of the LE-reconstruction procedure for three systems whose common feature is the existence of a slow degree of freedom. For the three cases we have a long time series of the velocity of the slow particle and, assuming that it can be fairly approximated by a Markov process, we infer the parameters of a LE. Characteristic properties of the reconstructed model—such as the stationary probability density function, the autocorrelation or power spectrum, and the mean squared displacement—are then computed analytically or by numerical simulations, and the results are compared with the original data.

*Harmonic chain* The first system we consider is a chain of 2N+1 coupled harmonic oscillators with Hamiltonian

(25) $$H=\frac{P^{2}}{2M}+\sum_{i=\pm 1,\ldots ,\pm N}\frac{p_{i}^{2}}{2m}+\frac{k}{2}\sum_{i=-N}^{N+1}{\left(q_{i}-q_{i-1}\right)}^{2},\;\;q_{-N-1}\equiv q_{N+1}\equiv 0$$

in which the heavy particle, referred to as the *intruder*, occupies the central position ($Q\equiv q_{0}$). The parameter *k* represents the elastic constant, while *m* and *M* are the mass of the light particles and that of the intruder, respectively. We adopt fixed boundary conditions for the first and the last particles for computational reasons: they prevent an unbounded drift of positions caused by the conservation of total momentum.

This system can be solved analytically. In a slightly modified version it has been extensively studied since 1960, when Rubin and Turner, in their seminal works [82,83], showed that the behavior of the heavy particle could be approximated by a Brownian motion, under the assumption of canonically distributed initial conditions. In particular, for the autocorrelation function of the heavy particle’s velocity C(t) one has

(26) $$C\left(t\right)∼\exp\left(-\frac{2\sqrt{km}}{M-m}t\right)+O\left(\frac{m}{M}\right)$$

when M/m≫1. Further analyses [100] on the frequency spectrum of the normal modes pointed out that the previous approximation was valid only if the ratio $M/\sqrt{km}$ continued to be finite when the heavy mass limit was taken. Several generalizations of this simple model have been explored: The linear chain with nearest-neighbors interactions has been shown to be just a particular case of a wider class of harmonic systems with similar properties [101,102], that can be used as “thermal baths” for the intruder even if the heavy particle is subjected to non-linear forces [84]. Further details on the numerical protocol and its application in this specific case can be found in [90].

**Figure 5:**
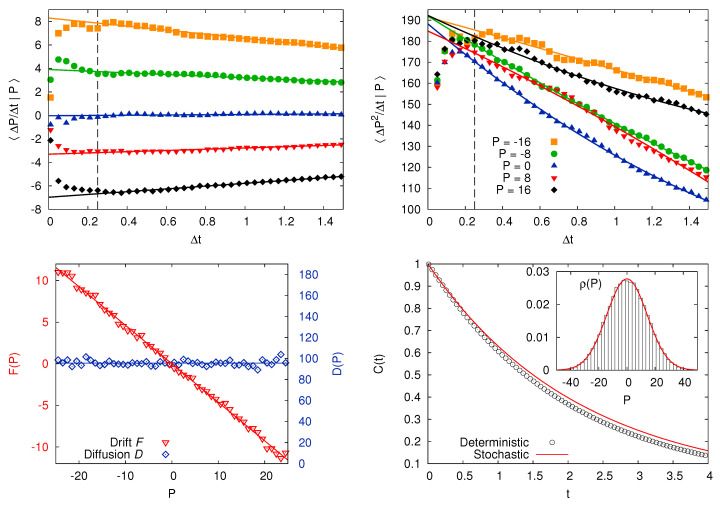
Harmonic chain. The model is Equation (25) with M=200, k=2500, 2N=2000, β≃1.0; integration step $\delta t=10^{-3}$. Top: Extrapolation of the limits (23) in order to estimate drift (**left**) and diffusivity (**right**), for several values of *P*. Bottom left: Drift and diffusion coefficients for the Langevin Equation describing *P*, inferred from simulations. Bottom right: Autocorrelation function for the velocity $\dot{Q}$ of the intruder; black circles represent the outcomes of molecular dynamics simulations, solid red line is computed by simulating LE with the previously inferred coefficients. Inset: Momentum p.d.f. from the same deterministic (histogram) and stochastic (solid red line) simulations.

The procedure is perfectly successful in reconstructing the parameters of the Langevin Equation from the data, as can be seen in Figure 5.

*Elastic gas* As second model we consider a 2D gas of elastic hard disks with a very low fraction of occupied volume. We simulate N=1000 disks: Of these 999 have mass m=1 and 1 has mass M=100 (the “tracer”). All the disks have diameter d=2r=0.01. They move with periodic boundary conditions in a square of area A=L×L=10. The number density is n=N/A=100, the coverage (or “packing”) fraction is $\phi =\pi *N*r^{2}/A=0.785\%$, i.e., the system is very dilute. We initialize the system with total kinetic energy K=1000 which is exactly conserved by the dynamics. This implies a kinetic temperature $T=\langle v_{i}^{2}\rangle /2=M\langle v_{0}^{2}\rangle /2=1$ with i≠0.

For a massive tracer in a dilute gas the dynamics of the Cartesian components of the tracer’s velocity $v_{0}\equiv \left(V_{x},V_{y}\right)$ follow two independent linear LEs i.e., Equation (22) with F(v)=−γv and $v=V_{x}$ or $v=V_{y}$. The damping coefficient is given by (see for instance [78,80,81])

(27) $$\gamma =2\sqrt{2\pi }nd\sqrt{\frac{T}{m}}\frac{m}{M}=\frac{2\sqrt{2\pi }}{100}\approx 0.05.$$

Finally the noise amplitude is determined by the Fluctuation-Dissipation theorem D=γT/M [103].

Performing the extrapolation procedure, we find that the reconstructed damping force can be described, as expected, as a linear function of the velocity, while the diffusivity is almost constant. Upon comparing the inferred parameters to the theoretical predictions discussed above, one finds a very good agreement (relative difference ∼1% for γ, ∼5% for $\sqrt{2D}$).

**Figure 6:**
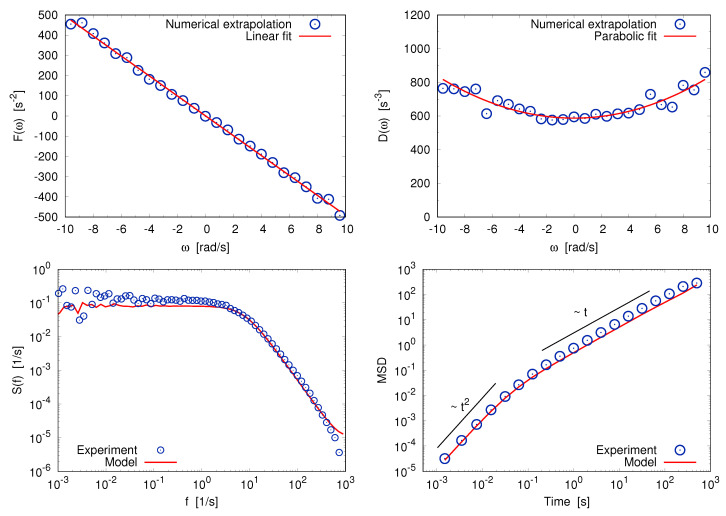
Dilute granular experiment. Top: Reconstructed drift (**left**) and diffusivity (**right**) for the angular velocity, fitted trough a linear function and a quadratic polynomial, respectively. Bottom: Power spectrum (**left**) and mean square displacement (**right**); experimental data (blue circles) are compared to those obtained by simulating the inferred stochastic model (red lines).

*Granular system* For the two previous examples we have a good theoretical understanding, therefore the numerical procedure does not reserve much surprises. More interesting is to use the procedure on *experimental* data of a system which lacks a well established theoretical understanding, for instance when far from equilibrium. The analyzed time series is that of the angular velocity of a rotator suspended in a vibrofluidized granular medium [104]. The granular medium made of N=300 spheres of diameter d=4 mm is placed in a cylindrical container of volume ∼7300 times that of a sphere (the average packing fraction is therefore ∼4%). The container is vertically shaken with a signal whose spectrum is approximately flat in a range $\left[f_{min},f_{max}\right]$ with $f_{min}=200$ Hz and $f_{max}=400$ Hz. A blade, our “massive tracer” with cross section ∼8d×4d, is suspended into the granular medium and rotates around a vertical axis. Its angular velocity ω(t) and the traveled angle of rotation $\theta \left(t\right)=\int_{0}^{t}\omega \left(t^{\prime }\right)dt^{\prime }$ are measured with a time-resolution of 2 kHz. The blade, interacting with the spheres, performs a motion qualitatively similar to an angular Brownian motion. The shaking intensity is measured by the normalized mean squared acceleration of the vibrating plate $\Gamma =\sqrt{\langle {\left(\ddot{z}\right)}^{2}\rangle }/g∼40$. Again, in view of the diluteness of the system and the large mass ratio between the tracer and the particles, the dynamics of ω(t) is expected to be well reproduced by a Markov process. In Figure 6 we show that the damping force is proportional to the angular velocity, as in the previous cases, while the reconstructed diffusivity presents a parabolic dependence on ω. Such multiplicative stochastic process can be numerically simulated and compared to the experimental data.

The three examples above concern situations where, even if not totally trivial, it is highly expected that a Markovian continuous model (a LE) provides an effective description. More in general, however, the guess that *v* is described by a Markovian process is not trivial at all. For instance, the experiment discussed in the third case above has been conducted in much more dense granular setups, for which there are several indications that the dynamics of the tracer is not Markovian [104,105]. Basically we have that the variable θ(t) is not able to describe in a good way the dynamics of the tracer and it is necessary to introduce (at least) another variable. Even the identification of such a variable is not obvious, this is part of a general problem which has been stressed by Onsager and Machlup in their seminal work on fluctuations and irreversible processes [106], with the caveat: *How do you know you have taken enough variables, for it to be Markovian?* In a similar way, Ma noted that [103]: *The hidden worry of thermodynamics is: We do not know how many coordinates or forces are necessary to completely specify an equilibrium state.*

## 6. Conclusions and Final Remarks

After a tribute to two classical models which had a seminal role in science, namely the Lorenz model and the Lotka-Volterra equations, we discussed the problem of model building and prediction with a data-based approach. A critical analysis shows the severe limits of such an approach and the necessity to use, in a clever way, models. Many interesting systems have a multiscale structure, i.e., there are different variables with very different characteristic times. While such a feature is at the origin of severe difficulties, it also opens the possibility to reduce the complexity by building an effective equation for the slow variables. In this respect we discussed the methodology and the difficulties to build effective equation for proteins, as well as mesoscopic descriptions based on the Langevin equation. Reviewing some of the methodologies used in the building of different models, it is well clear that there are no systematic protocols and, on the contrary it is always necessary to use the previous understanding of the considered system and, often, analogy and intuition [107,108].

Another important point in model building, perhaps the most difficult one, is to choose the “right” variables. This aspect, too often overlooked, was touched while discussing the Langevin equation, and underlined by Onsager and Machlup in their seminal paper [106] and by Ma in his book on the foundation of statistical mechanics [103]. The ability of choosing the “right” variables typically requires a conceptual abstraction which is key to scientific discoveries. For instance, in the ’80s, some researchers in the field of artificial intelligence (AI) devised BACON, a computer program to automatize scientific discoveries [109] (after the philosopher Francis Bacon who has been the champion of an inductive approach to science). Apparently, BACON was able to “discover” the Black’s law for temperature of a mixture of two liquids, the Snell’s law in optics, and the third Kepler’s law. Looking at the details of the procedure used by BACON, however, it seems difficult to conclude that naive AI methods can replace the traditional creative approach to scientific discoveries. Indeed, in the case of the third Kepler’s law, BACON used as input the numerical values of distance from the Sun, *D*, and revolution period, *P*, of planets. The program, then, discovered that $D^{3}$ is proportional to $P^{2}$. It is unfair to say that this represents a direct inductive approach only from data: The raw observables are not *D* and *P*, but a list of planetary positions seen from the Earth at different times. In his discovery, Kepler chose the “right” variables *D* and *P* as he was guided by strong beliefs in mathematical harmonies as well as the (at that time) controversial theory of Copernicus [110].

We conclude with some general considerations about recent ideas on the role of models and data in science. In the last centuries the scientific tradition had been based on two general pillars: A theoretical one grounded on mathematics and experimental methods. After World War II, numerical simulations surged as a third pillar for scientific investigations. In the most interesting cases, simulations are not a mere way to solve difficult mathematical problems but can reach the status of experiments that can trigger new theoretical developments. For example, the presence of slow decay in the molecular velocity autocorrelation in liquids (hydrodynamic tails) has been first observed in molecular dynamics simulations and then theoretically explained [111].

In the last decade, the possibility of extracting knowledge by data mining (i.e., through the algorithmic analysis of large amounts of data) seems to suggest the emergence of a fourth paradigm, a new scientific methodology to be added to the three already existing [112]. Ten years ago Chris Anderson, then the chief editor of the influential technology magazine *Wired*, published an article entitled “The End of Theory: The Data Deluge Makes the Scientific Method Obsolete” [113]. This article quickly became an ideological manifesto of datacentric enthusiasm supporting a general philosophy starting from “raw data”, without constructing modeling hypotheses and, therefore, without theory. We think that the surveyed procedures and, in particular, the difficulties of model building justify some skepticism about the claim that we are facing a new scientific revolution, the datacentric one. However, it should be acknowledged that recent results based on artificial intelligence (machine learning), as discussed at the end of Section 3.1, applied to data seem to support, at least in certain cases, the possibility either to construct mathematical models or to improve their predictability [17,18,21,22]. Nevertheless, two remarks are in order. The first one is that the above methods require the knowledge of the state variables. Second, one has to restrict a priori the class of mathematical structures (e.g., local interactions, degree of nonlinearity, and symmetries, etc.) and this cannot be done automatically without prior knowledge. Therefore, we can fairly conclude that such approaches can be powerful tools at a technical level but are not free from the key difficulties of model building. 

## Appendix A. An Attempt to Build Clever Low Dimensional Sysstems

This Appendix briefly presents an interesting method that combines mathematical models (i.e., the equations which are assumed to be known) and experimental data to derive clever low dimensional models. This can be seen as a way to improve standard Galerkin methods, such as that discussed in Section 2.1 for deriving the Lorenz model.

In the presence of coherent structures, sometimes, the dimension of the system is not too large so one can hope that few variables are enough for a good description. On the other, although from a mathematical point of view the choice of the functions $\left\{\phi_{n}\right\}$ is arbitrary, if one wants to use moderate values *N* it is necessary to use a “clever” complete, orthonormal set of eigenfunctions [8].

Of course one has to pay a price: The $\left\{\phi_{n}\right\}$ must be selected according to the specific dynamical properties of the problem under investigation, and, in addition it is necessary to experimental or numerical data. Let us discuss the method called proper orthonormal decomposition (POD), which allows to derive clever low-dimensional systems.

The procedure is rather elaborated, in few words: One has to find the eigenvalues and eigenfunctions of the integral equation

$$\int R\left(x,x^{\prime }\right)\phi_{n}\left(x^{\prime }\right)dx^{\prime }=\lambda_{n}\phi_{n}\left(x\right)$$

where the kernel $R(x,x^{\prime })$ is given by the spatial correlation functions, which must be computed by experimental (or numerical) data. For instance, assuming, just for sake of notation simplicity, that ψ is a scalar field one has $R\left(x,x^{\prime }\right)=\langle \psi \left(x\right)\psi \left(x^{\prime }\right)\rangle$. The field ψ(x,t) is thus reconstructed using this set of function and the expansion (2). The eigenvalues $\left\{\lambda_{n}\right\}$ are ordered in such a way to ensure that the convergence of the series is optimal, i.e., this expansion is the best approximation in $L_{2}$ norm.

Essentially, the POD procedure is a special case of the Galerkin method which captures the maximum amount of $L_{2}$ norm among all the possible truncations with *N* fixed. Such an approach has been successfully used to model different phenomena such as, e.g., jet-annular mixing layer, 2D flow in complex geometries and the Ginzburg-Landau equation [8,114].

We conclude by stressing that POD is not a straightforward procedure as thoughtful selections of truncation and norms are necessary in the construction of convincing low-dimensional models.
